# Special Issue "Plant Hormone Signaling"

**DOI:** 10.3390/ijms262110554

**Published:** 2025-10-30

**Authors:** Kinga Dziurka, Maria Duszyn, Ewa Muszyńska

**Affiliations:** 1 Department of Biotechnology, The Franciszek Górski Institute of Plant Physiology, Polish Academy of Sciences, Niezapominajek 21, 30-239 Kraków, Poland; 2 Department of Plant Genetics, Breeding and Biotechnology, Institute of Biology, Warsaw University of Life Sciences-SGGW, Nowoursynowska 159, 02-776 Warsaw, Poland; maria_duszyn@sggw.edu.pl; 3 Department of Botany, Institute of Biology, Warsaw University of Life Sciences-SGGW, Nowoursynowska 159, 02-776 Warsaw, Poland; ewa_muszynska@sggw.edu.pl

## 1. Introduction

Phytohormones play a key role in regulating every life process of the plant, at every stage of ontogeny. On the one hand, they allow the plant to fulfil its own development program, and on the other, to adapt to changing environmental conditions. They participate in the reception of stimuli and subsequent signal transduction, maintaining homeostasis and regulating the expression of relevant genes. In the complex network of plant metabolism, they do not act alone; rather, they interact synergistically or interdependently with other molecules, integrating and coordinating developmental processes under normal and stressful conditions. All these aspects are reflected in contemporary research on hormonal signaling in plants. The Special Issue entitled “Plant Hormone Signaling” focuses on the current state of knowledge and future prospects in this scientific research area. It is dedicated to the role of hormones in plant development processes such as: embryogenesis, reproduction, and formation of adventitious roots. Additionally, it addresses how plants adapt to abiotic (cold and drought) and biotic (pathogenic fungi and insects) stress conditions. The research undertaken concerns both the model plant *Arabidopsis thaliana* and the cultivated species: maize, wheat, oat, oilseed rape, sorghum and rhubarb or even ornamental fern—*Platycerium bifurcatum*.

## 2. Hormonal Control of Typical Plant Growth and Development

The life cycle of vascular plants is characterized by the alternation of two generations—the sporophyte (2n, diploid) producing haploid spores and the gametophyte (n, haploid) arising from these spores to generate gametes. This section describes the latest insights into the actions of different phytohormones in orchestrating processes that take place during both these generations to ensure undisturbed plant growth and reproduction.

Oliwa et al. [1] study focused on morpho-anatomical, hormonal and physiological changes occurring in four stages of sporulation within a single leaf of the fern, commonly known as the elkhorn fern (*Platycerium bifurcatum*). The results revealed a significant temporal and spatial distinction between sporophylic and trophophilic parts of the leaf with respect to pigment composition, flavonoid content and tissue lignification. For the first time, thermogenesis associated with spore formation and maturation was noticed, indicating a high level of metabolism in sporulating tissues. Furthermore, both parts of sporotrophophylls also varied in the accumulation of endogenous phytohormones. In sporulating areas, elevated levels of certain gibberellins (GA3, GA4, and GA6), auxin (IBA, i.e., indole-3-butyric acid), abscisic acid (ABA) and their glucosyl ester were detected, whereas in the non-sporulating areas, fluctuation of phytohormones occurred. The obtained results provide valuable information on which specific phytohormones support spore development, maturation and preparation for dormancy, as well as present a coherent and novel understanding of the metabolic activity of the functionally specialized parts of the same leaf during sporogenesis. In turn, the review paper of Yu et al. [2] illustrated the specification and spore formation in higher plants on the example of *Arabidopsis thaliana*. The authors demonstrate that auxin homeostasis precisely directs the transition of somatic cells into both male and female germ cells (called micro- and megaspore mother cells, respectively). The novelty of this work lies in the integration of germline specification into a coherent theoretical model encompassing auxin biosynthesis (particularly the TAA1/TAR2-YUCs), polar transport (PIN1 and other PINs), and signal transduction (via the TIR1/AFB-ARF and alternative mechanisms ABP1-TMK). A central place in this network is played by the transcription factor SPL/NZZ, which acts both as an effector of the auxin signal and as a feedback regulator controlling auxin biosynthesis and transport. The work also highlights the link between spatially restricted auxin accumulation and microRNA160, which negatively regulates the expression of ARFs, ensuring the selection of a single megaspore mother cell in the ovule. An important element of this review is also the emphasis on practical applications for plant breeding, indicating that the manipulation of key genes of auxin pathways can be used to improve crop fertility or produce seedless varieties.

While the above-mentioned review focuses on the determination of germline cells crucial for gametophyte formation, enabling sexual reproduction, the work of Dziurka et al. [3] provided an experimental extension of these processes on embryogenesis, showing that the auxin metabolites’ accumulation regulates the rate and quality of embryo development. In this research, the authors used 21-day-old haploid oat embryos produced after cross-pollination with maize and compared their auxin profile with zygotic embryos of various ages ranging between 1 to 21 days after pollination (DAP). It was found that 21-day haploid embryos contained half the amount of indole acetic acid (IAA) as their zygotic counterparts, with significantly higher amounts of inactive auxin forms (meIAA, IAA-Glu, IAA-Asp). Additionally, the microscopic visualization of IAA in zygotic embryo cells increased with time and became more concentrated in differentiating regions (e.g., shoot or root apical meristems, coleoptile), while the IAA signal in haploids was weaker and more diffuse. This innovative and multi-levelled study, combining quantitative and qualitative analyses with the microscopic imaging method, allowed the authors to conclude that disturbed auxin homeostasis is consistent with delayed morphogenesis, and 21-day haploid embryos correspond to a stage of zygotic ones earlier than 9 DAP. From the practical perspective of Dziurka et al. [3] findings, the targeted and dynamic modulation of auxin may offer a strategy to enhance the success of haploid embryo rescue procedures. Then, the effects of auxin on the subsequent post-embryonic stages of plant development are demonstrated by Yang et al. [4]. Authors revealed that the maize NAC transcription factor ZmNAC17 positively regulates mesocotyl elongation by modulating auxin biosynthesis and reactive oxygen species (ROS) homeostasis. Loss-of-function mutants exhibited shorter mesocotyls and decreased IAA levels, together with increased ROS accumulation in comparison to wild-type. Moreover, transcriptomic analysis identified differentially expressed genes enriched in tryptophan metabolism and antioxidant activity pathways, implicating modulations in auxin and ROS-related gene expression. Physiological assays confirmed altered activities of antioxidant enzymes, such as catalase and peroxidase in mutants, linking ROS imbalance to inhibited mesocotyl growth. These findings suggest that ZmNAC17 integrates auxin signaling and redox regulation to control early maize seedling development, providing valuable insights for improving seedling emergence and stress resilience. In turn, Wojtania et al. [5] studied the effects of IBA and its interaction with ethylene (ET) on adventitious root formation in culinary rhubarb, which is known for its irregular and ineffective rooting in vitro. The authors showed that the addition of a precursor of ET biosynthesis to the IBA-enriched medium increases rooting frequency to nearly 97%. Mechanistic insights revealed that ET altered endogenous hormonal balance by decreasing cytokinin and auxin levels at the shoot base. This is accompanied by an increase in the expression of genes encoding: a transcriptional repressor in the early auxin response (*IAA17*), a synthetase that converts active IAA into inactive conjugates (*GH3.1*), and a gene involved in ABA catabolism (*CYP707A1*). The above mentioned changes were coincident with enhanced rooting response. Contrary, the blocking of ET production and action triggered JA and ABA accumulation as well as upregulation of genes responsible for ABA biosynthesis and signaling (*PP2C49*, *CBF4*) that drastically reduced adventitious root formation. The identified hormonal cross-talk opens the way for optimal multiplication of rhubarb, enabling reliable production of bioactive-rich, elite plant material.

Although in adventitious rooting, ABA emerged as a negative regulator, it plays an opposite role during seed and fruit formation, which is reviewed in detail by Zheng et al. [6]. It is summarized that, in the seed phase, elevated ABA levels help maintain dormancy, while during fruit development, this phytohormone regulates pigment synthesis and sugar accumulation. The review also discusses an exogenous ABA application strategy, involving the targeted use of ABA at various stages of the plant life cycle, including seed storage, pre-sowing treatment, and seed production, to optimize germination performance and overall crop quality. Furthermore, it highlights three main strategies for the agricultural application of ABA: the use of ABA analogues, the development of ABA signal modulators (including enhancers and inhibitors), and breeding techniques based on ABA signaling. Collectively, these strategies aim to enable precise regulation of plant development, enhance stress tolerance, and increase crop yield, representing an innovative approach to modern agriculture.

## 3. Plant Hormone Signaling in Abiotic Stresses

Stress physiology is a particularly pressing research issue. The changing climate not only creates inhospitable, difficult habitats, forcing plants to adapt and activate processes that maintain homeostasis and allow them to survive, but also influences the shifting ranges of pathogens, which forces plants to develop immune responses. All these processes are based on regulatory mechanisms in which hormones, as basic signaling molecules, are directly involved.

Ryś et al. [7] studied hormonal and molecular changes in the leaves of two winter oilseed rape cultivars, ‘Kuga’ and ‘Thun’, which accompanied the cold acclimation and deacclimation process. The increase in the content of plant growth-promoting gibberellins (both precursors and active forms), as well as IAA-Asp and IAA-Glu auxin conjugates with a simultaneous decrease in ABA during deacclimation, indicates that plants are getting ready to resume growth. This is accompanied by upregulated expression of the gene encoding auxin transcription factor *BnARF*. In turn, the increase in *BnABF2* (transcription factor of ABA signaling) gene expression despite the decrease in ABA content during deacclimation may be part of a strategy preparing the plant for reacclimation if the ambient temperature drops again. Stachurska et al. [8] explored the role of brassinosteroids in the deacclimation process. The reduced expression of *BRI1*, encoding the brassinosteroid receptor, correlated with the lower content of the protein BRI1 that occurs during cold acclimation, is reversed during deacclimation. This is accompanied by upregulated expression of *SERK1* (encoding a protein involved in brassinosteroid signaling). The opposite trend is presented by the relative expression of the *COR14* (a gene involved in the response of plants to low temperature), induced by brassinosteroids. The observed changes suggest the participation of the studied factors in the mechanisms regulating the plant’s adaptation to changing environmental temperature. Deacclimation reduces the frost resistance of rapeseed, but as the authors prove, this can be partially prevented by spraying with steroid regulators, e.g., EBR. However, this phenomenon is cultivar-dependent, and it is probably related to changes in cell membrane permeability. The practical aspect of the authors’ research is to determine markers of the deacclimation process, such as: leaf reflectance, chlorophyll a fluorescence, and gas exchange. It is worth emphasizing that changes in the above-mentioned parameters can be examined in a non-invasive manner. Studying the changes in the leaf light reflectance spectrum in the 500–650 nm range may be particularly valuable to assess the deacclimation occurrence over large cultivated areas using satellite monitoring.

The increase in average temperatures causes the soil to dry out faster, which, combined with limited rainfall and snowfall, contributes to the occurrence of droughts. The study of hormonal regulation of adaptation mechanisms to drought stress is a current research direction, especially in the face of ongoing climate change. Laskoś et al. [9] studied the physiological condition of two wheat cultivars, CS and SQ1, after 14 days of drought. In both cultivars, reduced net photosynthesis, transpiration and stomatal conductance were observed under the influence of drought, which is usually the result of ABA accumulation. Indeed, the CS variety showed significant ABA accumulation compared to the control. An interesting response was observed in SQ1, where the observed physiological changes were not accompanied by changes in ABA levels. The authors hypothesized a possible redistribution of ABA to the epidermis. Another possibility is that the ABA peak occurred earlier than the 14th day of drought, and therefore, physiological responses to this stimulus, and the lack of changes in ABA concentration can be observed. The smaller reduction in net photosynthesis and water loss in the SQ1 variety suggests that this cultivar tolerates water deficits better than CS variety. This is justified by the origin of these cultivars, as they were originally selected to differ ABA accumulation under drought stress (CS-low, SQ1-high). The above mentioned studies have application potential because they indicate biochemical or physiological parameters that may be markers of stress in individual plants or large-scale crops.

## 4. Interactions of Phytohormones in Plant Defense Against Biotic Stress

Similar to their role in abiotic stress adaptation, phytohormones regulate plant defenses against biotic stresses by integrating complex signaling networks, ensuring precise and effective protection against pathogens and herbivores.

Paclobutrazol-Resistance (*PRE*) genes encode a small group of atypical basic helix–loop–helix (bHLH) transcription factors that counteract the growth regulator paclobutrazol by inhibiting gibberellin synthesis and thereby modulating diverse plant developmental processes. PRE proteins interact with typical bHLH transcription factors to regulate downstream genes, thus influencing growth, hormonal signaling, and responses to environmental stimuli such as light, temperature, and stress. Guo et al. [10] conducted a comprehensive genome-wide identification and functional characterization of seven *PRE* homologs (*SbPRE* genes) in sorghum. Analysis of cis-regulatory elements within *SbPRE* promoters indicated enrichment for hormone- and stress-responsive motifs. Furthermore, several *SbPRE* genes, especially *SbPRE4*, demonstrated inducible expression under both abiotic stress and aphid infestation. Functional analysis using transgenic Arabidopsis lines overexpressing *SbPRE4* provided evidence that the gene promotes aphid resistance through shifting jasmonic acid (JA) biosynthesis and signaling towards its accumulation, highlighting its role in herbivore defense. These findings indicate that *SbPRE* genes, particularly *SbPRE4*, contribute to the aphid stress response and provide a foundation for future studies on their biological and molecular functions, offering valuable insights for the understanding and application of *PRE* family genes in sorghum and other plant species. Another regulator of JA signaling revealed by Falconieri et al. [11] is glyoxalase I 4 (GLYI4), which is not only a pivotal component in the detoxification of cytotoxic aldehyde methylglyoxal (MG), but also changes JA biosynthesis and signaling pathways, introducing a new element into the molecular network underlying plant resistance to biotic stress. In Arabidopsis, loss-of-function *glyI4* mutants exhibited stunted growth, reduced rosette diameter, altered chlorophyll and carotenoid composition, and elevated reactive oxygen species levels, linked with impaired MG detoxification and enhanced oxidative stress. Moreover, gene expression analyses indicated disturbances in the regulation of the JA pathway, including downregulation of biosynthesis and signaling genes, along with increased expression of repressors, which is associated with weakened activation of defense mechanisms. Consistent with these molecular alterations, disease resistance bioassays using the necrotrophic pathogen *Botrytis cinerea* demonstrated a modified resistance pattern in *glyI4* mutants, with MeJA-induced protection being less effective compared to wild-type plants. Furthermore, treatment with exogenous MG exacerbated the diminished defense responses and plant condition, confirming the negative impact of this metabolite on JA pathway functionality and oxidative stress balance. These findings point GLYI4 as a link connecting MG detoxification, oxidative stress control, and proper hormonal JA signaling function, which together determine effective plant defense against necrotrophic pathogens. This study broadens our understanding of the complex molecular mechanisms underlying plant adaptation to biotic stress and offers potential avenues for improving disease resistance in crop plants. Among other proteins modulating jasmonate-dependent defense, TIRP is proposed to be involved in protecting plants against biotic stress. In a recent study, Neufeld et al. [12] examined the role of the Arabidopsis *TIRP* gene, encoding a TIR-X protein, in defense against necrotrophic and hemibiotrophic fungal pathogens (*Alternaria brassicicola* and *Verticillium dahliae*, respectively), as well as polyphagous insect *Spodoptera littoralis*. Using knock-out and overexpressing Arabidopsis lines, researchers assessed TIRP’s influence on phytohormone-mediated defense responses, focusing on salicylic acid (SA) and JA pathways, along with overall plant resistance. The study revealed that while *TIRP* expression is induced upon pathogen attack, it does not regulate the levels of jasmonates or SA during these interactions. Moreover, mutation or overexpression of *TIRP* did not significantly alter plant resistance to the tested fungal pathogens or herbivory, as measured by pathogen growth and insect performance assays. The results suggest that TIRP, even if involved in defensive responses to biotic stress, does not act through hormonal regulation. Presented studies advance our understanding of phytohormone interactions in plant biotic stress defense, revealing novel regulators of JA signaling like SbPRE4 and GLYI4, while also cautioning that not all immune-related proteins, such as TIRP, are hormone-dependent. They underscore the complexity and specificity of hormonal crosstalk in plant resilience to pathogens and pests.

## 5. Conclusions and Future Perspectives

Our Special Issue entitled “Plant Hormone Signaling” covers a wide range of issues related to plant hormone involvement in growth, reproduction, and stress adaptation across diverse species. The published studies not only expand our current insight into the complex modes of phytohormone actions but also demonstrate the existing gaps and future research aspects that remain to be discovered. We already know a lot about hormonal regulation in plants, but this mainly concerns the so-called active forms of these compounds. The functions of hormone conjugates, which are often abundant in plants and are generally considered inactive or even permanently deactivated forms of hormones, remain unknown. The complex network of cross-talk not only between different phytohormones but also between phytohormones and other metabolites also awaits exploration. Similarly, as is the understanding of plant-plant, plant-microbes, or plant-pests hormonal communication. We see the need to develop new, including non-invasive, methods of measuring or visualizing phytohormones in plant tissues. Moreover, in the era of high-throughput analyses, machine learning and artificial intelligence, it would be desirable to create a model of the complex network of phytohormone connections at various levels of regulation. While appreciating the potential of regulatory substances, we also look forward to implementing research results in agricultural production to improve and protect crops.
